# A Severe Cold

**Published:** 1880-01

**Authors:** 


A Severe Cold can be soonest cured by remaining within doors, in a warm room and near the fire, until all signs of it have disappeared. Then, care should be taken to prevent a relapse by having the feet warmly clad, and the whole body, and particularly the chest and the back of the neck, well protected when going out.

